# Supplementary material for: Country-level instability is related to a stronger perceived climate of polarization across 44 countries
Source: Commun Psychol. 2026 Feb 25;4:63. doi: 10.1038/s44271-026-00422-x (PMC13046976; doi:10.1038/s44271-026-00422-x)
Supplement: Supplementary file 2 — Supplementary Materials [file 44271_2026_422_MOESM2_ESM.pdf]

Supplementary Information

Supplementary Table 1. Sample Details by Country

Country	Country Code	*N*	Language	Age range (*M*, *SD*)	Mean economic PO (*SD*)	Mean social PO (*SD*)	Mean SES (*SD*)	Mean religiosity (*SD*)
Argentina	24	203	Spanish	18-78 (44.98, 15.73)	4.25 (1.54)	4.07 (1.55)	3.81 (1.01)	3.37 (1.94)
Australia	17	201	English	18-88 (45.91, 17.22)	3.80 (1.47)	3.83 (1.58)	3.56 (1.18)	2.80 (2.12)
Austria	20	204	German	18-86 (46.77, 15.76)	3.86 (1.23)	3.85 (1.35)	3.80 (0.99)	2.68 (1.96)
Belgium	31	205	French, Dutch	18-80 (47.03, 16.30)	4.11 (1.50)	3.89 (1.60)	3.84 (0.96)	2.19 (1.62)
Brazil	25	200	Portuguese	18-85 (45.48, 16.01)	4.06 (1.90)	4.03 (1.96)	3.97 (0.99)	4.82 (2.08)
Bulgaria	44	201	Bulgarian	20-73 (46.65, 13.31)	3.91 (1.34)	4.24 (1.50)	3.23 (1.05)	3.77 (1.81)
Canada	28	203	English	18-77 (45.63, 15.98)	3.87 (1.71)	3.62 (1.78)	3.79 (1.17)	3.36 (2.23)
Chile	36	207	Spanish	18-84 (45.43, 14.31)	4.05 (1.74)	3.98 (1.80)	3.83 (0.96)	3.56 (2.15)
China	18	200	Simplified Chinese	19-70 (42.81, 14.27)	3.56 (1.70)	3.77 (1.78)	4.40 (1.14)	2.55 (2.02)
Colombia	26	202	Spanish	18-76 (42.78, 15.07)	4.34 (1.75)	4.37 (1.76)	3.76 (0.88)	4.66 (1.82)
Czech Republic	12	204	Czech	18-80 (45.36, 16.15)	4.12 (1.50)	4.02 (1.67)	3.78 (0.95)	1.92 (1.56)
Denmark	8	203	Danish	18-91 (49.55, 16.00)	4.00 (1.78)	3.93 (1.71)	3.89 (1.20)	2.42 (1.71)
Finland	33	200	Finnish	18-94 (45.98, 16.13)	3.74 (1.41)	3.66 (1.52)	3.08 (1.18)	2.22 (1.75)
France	3	200	French	18-88 (46.16, 16.83)	4.21 (1.64)	4.15 (1.71)	3.95 (0.96)	2.78 (1.89)

Germany	5	204	German	18-82 (47.45, 16.12)	3.91 (1.35)	3.93 (1.44)	3.99 (1.10)	2.32 (1.89)
Greece	10	204	Greek	19-74 (44.03, 15.21)	3.81 (1.42)	3.84 (1.66)	3.77 (1.12)	4.24 (2.03)
Hong Kong	38	202	Traditional Chinese	20-77 (43.54, 14.05)	3.93 (1.22)	3.95 (1.24)	3.96 (1.07)	3.23 (2.07)
Hungary	11	201	Hungarian	18-78 (47.39, 15.48)	4.16 (1.55)	4.05 (1.61)	3.42 (1.03)	2.69 (1.87)
India	21	200	English	18-78 (45.06, 16.16)	4.67 (1.60)	4.56 (1.72)	4.91 (1.19)	5.48 (1.72)
Indonesia	39	203	Indonesian	18-76 (39.76, 13.88)	4.42 (1.44)	4.55 (1.51)	4.36 (0.83)	6.42 (1.27)
Ireland	16	200	English	18-85 (45.42, 16.60)	3.76 (1.31)	3.74 (1.49)	3.79 (1.11)	3.29 (1.99)
Italy	4	201	Italian	18-84 (45.52, 15.99)	3.44 (1.53)	3.26 (1.62)	3.62 (1.18)	3.27 (1.98)
Japan	19	201	Japanese	18-89 (46.75, 17.03)	4.01 (1.03)	4.07 (1.16)	3.66 (1.11)	2.31 (1.57)
Mexico	29	202	Spanish	18-76 (44.56, 15.86)	3.96 (1.71)	3.74 (1.71)	4.13 (1.00)	4.00 (2.04)
Netherlands	32	213	Dutch	19-80 (49.69, 15.44)	4.10 (1.50)	4.02 (1.60)	3.68 (1.25)	2.91 (2.00)
New Zealand	34	201	English	18-87 (46.90, 17.16)	4.03 (1.59)	3.90 (1.77)	3.73 (1.17)	3.13 (2.35)
Norway	9	202	Norwegian	18-78 (47.60, 16.34)	4.10 (1.61)	4.00 (1.61)	3.98 (1.18)	2.73 (1.93)
Philippines	41	201	English	18-82 (45.34, 16.15)	4.82 (1.45)	4.80 (1.61)	3.71 (0.99)	5.77 (1.52)
Poland	13	200	Polish	18-78 (45.31, 15.99)	3.94 (1.63)	3.82 (1.86)	3.44 (1.17)	3.75 (2.01)
Portugal	27	204	Portuguese	18-81 (45.36, 15.80)	3.82 (1.49)	3.64 (1.68)	3.92 (0.83)	3.19 (1.87)
ROK	14	200	Korean	19-81 (45.46, 15.73)	3.88 (1.32)	4.09 (1.27)	3.46 (1.16)	3.03 (2.11)

Romania	42	201	Romanian	18-76 (47.23, 14.53)	3.87 (1.57)	3.97 (1.64)	3.35 (1.09)	4.15 (1.78)
Singapore	35	200	English	21-79 (45.58, 15.53)	4.17 (1.14)	4.22 (1.29)	3.96 (1.08)	4.22 (2.00)
Slovakia	43	202	Slovak	18-80 (48.26, 14.49)	3.95 (1.50)	3.79 (1.63)	3.75 (1.15)	3.44 (2.08)
South Africa	30	204	English	18-84 (44.95, 16.24)	4.47 (1.51)	4.42 (1.70)	3.93 (1.18)	5.04 (2.12)
Spain	6	202	Spanish	18-76 (45.85, 15.95)	3.62 (1.57)	3.37 (1.62)	3.88 (0.98)	2.72 (1.83)
Sweden	7	202	Swedish	18-81 (46.12, 16.42)	3.81 (1.64)	3.76 (1.82)	3.96 (1.26)	2.09 (1.73)
Taiwan	37	200	Traditional Chinese	20-78 (45.09, 15.58)	3.91 (1.44)	3.88 (1.64)	3.94 (1.05)	3.78 (1.84)
Thailand	15	209	Thai	18-76 (41.04, 14.12)	3.79 (1.47)	3.47 (1.46)	3.63 (0.94)	4.88 (1.78)
Türkiye	22	208	Turkish	19-74 (41.48, 14.25)	3.28 (1.79)	3.61 (1.87)	4.00 (1.04)	4.37 (2.18)
UAE	23	200	Arabic	20-95 (47.05, 18.85)	4.26 (1.47)	4.39 (1.57)	4.68 (1.08)	5.75 (1.38)
UK	1	201	English	18-78 (47.28, 16.56)	3.80 (1.48)	3.83 (1.56)	3.79 (1.24)	3.11 (2.30)
USA	2	204	English	18-84 (46.50, 16.82)	3.97 (1.81)	3.85 (1.90)	3.72 (1.30)	4.04 (2.16)
Vietnam	40	212	Vietnamese	19-69 (38.43, 12.57)	3.56 (1.42)	3.60 (1.56)	4.04 (0.84)	3.87 (2.10)

Note. PO = political orientation. SES = socioeconomic status. ROK = Republic of Korea. UAE = United Arab Emirates. UK = United Kingdom. USA = United States of America.

Supplementary Table 2. Comparison of Sample Gender Ratios Against Population-Level Statistics

Country	Sample % Female	Population % Female	Difference
Argentina	51.7	50.4	+1.3
Australia	51.2	50.4	+0.8
Austria	51.5	50.8	+0.7
Belgium	49.8	50.7	-0.9
Brazil	51.0	50.8	+0.2
Bulgaria	54.2	51.6	+2.6
Canada	50.7	50.3	+0.4
Chile	59.9	50.3	+9.6
China	51.5	49.1	+2.4
Colombia	51.5	50.7	+0.8
Czech Republic	52.0	50.7	+1.3
Denmark	53.7	50.3	+3.4
Finland	51.0	50.6	+0.4
France	51.5	51.5	+0.0
Germany	50.5	50.6	-0.1
Greece	51.5	51.6	-0.1
Hong Kong	52.5	55.0	-2.5
Hungary	54.7	52.0	+2.7
India	51.5	48.4	+3.1
Indonesia	49.3	49.8	-0.5
Ireland	51.5	50.5	+1.0
Italy	51.2	51.1	+0.1
Japan	51.2	51.2	+0.0
Mexico	51.0	51.5	-0.5
Netherlands	50.7	50.3	+0.4
New Zealand	51.7	50.3	+1.4
Norway	51.5	49.6	+1.9
Philippines	51.2	50.1	+1.1
Poland	51.5	51.6	-0.1
Portugal	51.5	52.4	-0.9
Republic of Korea	47.8	51.6	-3.8
Romania	51.5	48.3	+3.2
Singapore	62.4	51.2	+11.2
Slovakia	52.5	51.3	+1.2
South Africa	51.5	50.1	+1.4
Spain	51.5	50.9	+0.6
Sweden	51.0	49.6	+1.4
Taiwan	51.0	50.7	+0.3
Thailand	54.5	51.3	+3.2
Türkiye	47.6	50.1	-2.5
United Arab Emirates	52.5	36.1	+16.4
United Kingdom	51.7	50.8	+0.9
United States of America	51.0	49.8	+1.2
Vietnam	50.0	51.0	-1.0

Note. Population-level statistics were obtained from the World Bank Group (2025) and pertain to 2024 estimates. Population-level statistics for Taiwan were obtained separately from the Taiwanese Department of Household Registration (2025) and pertain to 2024 estimates. Mean difference = 1.4 percentage points, range = 0-16.4.

Supplementary Table 3. Survey Items

Item Label	Content	Response	Notes
Demographic Information			
Age	What is your age?		
Gender	Which gender do you identify as?	[1] Male<br>[2] Female<br>[3] Other (please state)<br>[4] Prefer not to say	
Religiosity	If you follow a religion, how important is that religion in your daily life?	[1] Not at all important<br>[2]<br>[3]<br>[4] Moderately important<br>[5]<br>[6]<br>[7] Extremely important	
SES_RealWealth	Relative to others in your country, how would you classify your own wealth?	[1] Very poor<br>[2]<br>[3]<br>[4] Average in wealth<br>[5]<br>[6]<br>[7] Very wealthy	
Econ_Conserv	Please indicate your political beliefs from left/liberal to right/conservative on issues of the economy (e.g., social welfare, government spending, tax cuts).	[1] Left/liberal<br>[2] 2<br>[3] 3<br>[4] 4<br>[5] 5<br>[6] 6<br>[7] Right/conservative	
Social_Conserv	Please indicate your political beliefs from left/liberal to right/conservative on social issues (e.g., immigration, homosexual marriage, abortion).	[1] Left/progressive<br>[2] 2<br>[3] 3<br>[4] 4<br>[5] 5<br>[6] 6<br>[7] Right/conservative	
Perceived Climate of Polarization			
[Introduction]	Thinking about the dominant groups of voters in your society, to what extent do you think the groups:		
Per_Pol_Dislike	Dislike one another?	[1] Not at all<br>[2]<br>[3]<br>[4]<br>[5] Extremely	
Per_Pol_Distrust	Distrust one another?	[1] Not at all<br>[2]<br>[3]<br>[4]<br>[5] Extremely	
Per_Pol_Distance	Distance themselves from one another?	[1] Not at all<br>[2]<br>[3]<br>[4]<br>[5] Extremely	

Anomie			
[Introduction]	Think of your society and indicate to what extent you agree or disagree with the following statements. In my society today…		The social fabric subscale is comprised of items 1-6.<br>The leadership subscale is comprised of items 7-12.
Anomie_1	People think that there are no clear moral standards to follow.	[1] Strongly disagree<br>[2]<br>[3]<br>[4]<br>[5]<br>[6]<br>[7] Strongly agree	
Anomie_2	Everyone thinks of himself/herself and does not help others in need.	[1] Strongly disagree<br>[2]<br>[3]<br>[4]<br>[5]<br>[6]<br>[7] Strongly agree	
Anomie_3	Most people think that if something works, it doesn't really matter whether it is right or wrong.	[1] Strongly disagree<br>[2]<br>[3]<br>[4]<br>[5]<br>[6]<br>[7] Strongly agree	
Anomie_4	People do not know who they can trust and rely on.	[1] Strongly disagree<br>[2]<br>[3]<br>[4]<br>[5]<br>[6]<br>[7] Strongly agree	
Anomie_5	Most people think that honesty doesn't work all the time; dishonesty is sometimes a better approach to get ahead.	[1] Strongly disagree<br>[2]<br>[3]<br>[4]<br>[5]<br>[6]<br>[7] Strongly agree	
Anomie_6	People are cooperative.	[1] Strongly disagree<br>[2]<br>[3]<br>[4]<br>[5]<br>[6]<br>[7] Strongly agree	Reverse scored
Anomie_7	The government works towards the welfare of people.	[1] Strongly disagree<br>[2]<br>[3]<br>[4]<br>[5]<br>[6]	Reverse scored

		[7] Strongly agree	
Anomie_8	The government is legitimate.	[1] Strongly disagree<br>[2]<br>[3]<br>[4]<br>[5]<br>[6]<br>[7] Strongly agree	Reverse scored
Anomie_9	The government uses its power legitimately.	[1] Strongly disagree<br>[2]<br>[3]<br>[4]<br>[5]<br>[6]<br>[7] Strongly agree	Reverse scored
Anomie_10	Politicians don't care about the problems of average person.	[1] Strongly disagree<br>[2]<br>[3]<br>[4]<br>[5]<br>[6]<br>[7] Strongly agree	
Anomie_11	The government laws and policies are effective.	[1] Strongly disagree<br>[2]<br>[3]<br>[4]<br>[5]<br>[6]<br>[7] Strongly agree	Reverse scored
Anomie_12	Some laws are not fair.	[1] Strongly disagree<br>[2]<br>[3]<br>[4]<br>[5]<br>[6]<br>[7] Strongly agree	

Supplementary Note 1

Construct validity of the Perceived Climate of Polarization scale

A reviewer raised concerns about whether the Perceived Climate of Polarization scale validly captures perceptions of polarization. To address this, we conducted construct validity analyses comparing the scale with three theoretically relevant measures that were included in the broader dataset:

1. **Perceived Moral Polarization** (Crimston et al., 2021). Participants were asked to think about the two major groups of people in their country with different sets of moral beliefs and opinions. They were asked to rate the extent to which they agree that the differences in moral opinions and beliefs between these groups is large on a scale from 1 (*Strongly disagree*) to 7 (*Strongly agree)*.
2. **Perceived Moral Overlap** (Crimston et al., 2021). Participants were asked to think about the two major groups of people in their country with different sets of moral beliefs and opinions. Using the graphics below as a guide, participants were asked to rate the extent to which they think the core moral values of the two groups overlap in their country. Responses were reverse coded so that higher values indicate greater dissimilarity between groups.

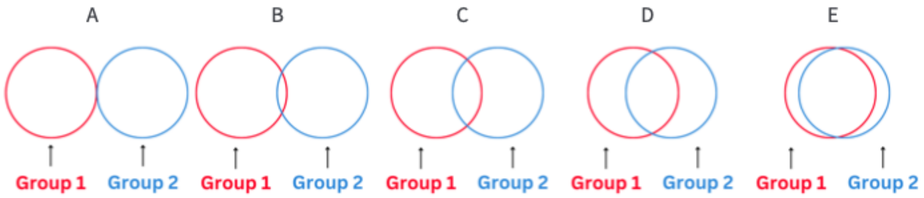

3. **Affective Polarization**. Participants were asked to think of people who hold (1) very similar and (2) very different opinions and beliefs to them on most issues. For both groups, participants were asked to rate how cold or warm their feelings are toward those people using a slider from 0 (*Cold*) to 100 (*Warm*). In line with other work using partisan feeling thermometers to measure affective polarization (e.g., Iyengar et al., 2012), a difference score was calculated by subtracting ratings for dissimilar others from ratings for similar others. Negative scores indicating greater warmth toward dissimilar others were excluded from analyses as they do not reflect the construct of affective polarization as it is typically defined (i.e., bias against partisan outgroups).

To examine convergent and discriminant validity, we calculated correlations between the variables of interest at three levels: (1) pooled individual-level correlations, (2) between-country correlations, and (3) within-country correlations.

Supplementary Table 4.1 shows the pooled, individual-level correlations between the variables. The Perceived Climate of Polarization was more strongly correlated with Perceived Moral Polarization and Perceived Moral Overlap than with Affective Polarization. This pattern suggests that the scale is more strongly related to other societal perception measures than to respondents' expressions of partisan bias.

Supplementary Table 4.1. Pooled Individual-Level Correlations

Variables	1.	2.	3.	4.
1. Perceived Climate of Polarization	—			
2. Perceived Moral Polarization	0.35***	—		
3. Perceived Moral Overlap	0.20***	0.25***	—	
4. Affective Polarization	0.18***	0.18***	0.10***	—

Note. * $p < .05$, ** $p < .01$, *** $p < .001$.

Supplementary Table 4.2 presents the between- and within-country correlations between the variables. At the between-country level, Perceived Climate of Polarization was more strongly and significantly correlated with Perceived Moral Polarization and Perceived Moral Overlap. It was not significantly correlated with Affective Polarization. At the within-country level, Perceived Climate of Polarization was most strongly correlated with Perceived Moral Polarization, consistent with our expectations. However, the scale was more strongly correlated with Affective Polarization than with Perceived Moral Overlap.

Supplementary Table 4.2. Between- and Within-Country Correlations

Variables	1.	2.	3.	4.
1. Perceived Climate of Polarization	—	0.32***	0.17***	0.19***
2. Perceived Moral Polarization	0.71***	—	0.23***	0.19***
3. Perceived Moral Overlap	0.65***	0.66***	—	0.11***
4. Affective Polarization	0.02	0.11	-0.03	—

Note. Between-country correlations are underneath the diagonal and within-country correlations are above. * $p < .05$, ** $p < .01$, *** $p < .001$.

Across three levels of analysis (pooled individual-level, between-country, and within-country), the Perceived Climate of Polarization scale was more strongly related to other perception-based constructs than to affective polarization. Although one measure (Perceived Moral Overlap) exhibited a weaker within-country association than expected, this discrepancy was limited to one variable at a single level of analysis. Overall, the broader pattern of results provides support for convergent and discriminant validity, suggesting that the scale is more strongly related to *perceptions* of the societal polarization than to expressions of partisan bias.

Measurement invariance of the Perceived Climate of Polarization scale

We tested measurement invariance of the Perceived Climate of Polarization scale in three steps. First, we fitted a configural model to the data – that is, a multi-group confirmatory factor analysis (MGCFA) model without restrictions on parameters. As shown in Supplementary Table 4.3, this model demonstrated excellent fit, indicating that the overall factor structure was comparable across countries. Next, we tested a metric invariance model, which constrained factor loadings across groups. The Comparative Fit Index (CFI) decreased by .006 but fell within the recommended threshold of .01 (Chen, 2007). Although changes in the Root Mean Square Error of Approximation (RMSEA) and Standardized Root Mean Squared Residual (SRMR) exceeded conventional thresholds, these indices are known to be

sensitive to model complexity, which can disadvantage more parsimonious models (Putnick & Bornstein, 2017). We therefore concluded that the data provided sufficient support for metric invariance. Finally, we tested a scalar invariance model, which constrained both factor loadings and intercepts across countries. This model did not satisfy conventional fit criteria (Chen, 2007) and thus scalar invariance was not supported.

Supplementary Table 4.3. Measurement Invariance

	CFI scaled	RMSEA scaled	SRMR	Chi-square	ΔCFI scaled	ΔRMSEA scaled	ΔSRMR
**Configural**	1.000	0	0	0			
**Metric**	0.994	0.057	0.039	152.690	-0.006	0.057	0.039
**Scalar**	0.976	0.084	0.055	427.839	-0.018	0.027	0.016

Supplementary Table 5. Linear Mixed Models Examining the Effects of Country-Level Variables on the Perceived Climate of Polarization, Perceived Breakdown of Leadership, and Perceived Breakdown of Social Fabric (Without Demographic Controls)

	Perceived Climate of Polarization					Perceived Breakdown of Leadership					Perceived Breakdown of Social Fabric				
**Predictors**	***N***	**β**	***df***	**95% CI**	***p***	***N***	**β**	***df***	**95% CI**	***p***	***N***	**β**	***df***	**95% CI**	***p***
**Economy and inequality**															
GDP PPP per capita	8652	-0.13^	41.01	[-0.22, -0.04]	.005 **	8564	-0.09^	40.99	[-0.23, 0.05]	.196	8544	-0.12^	41.01	[-0.21, -0.03]	.011 *
Income inequality	8054	0.12^	38.01	[0.03, 0.21]	.011 *	7972	0.14^	37.99	[0.00, 0.27]	.058	7954	0.14^	38.01	[0.06, 0.23]	.002 **
Unemployment	8851	0.14^	42.01	[0.06, 0.23]	.002 **	8758	0.20	41.97	[0.07, 0.32]	.004 **	8741	0.17	41.99	[0.09, 0.25]	< .001 ***
Youth NEET	8453	0.15	39.99	[0.06, 0.24]	.002 **	8367	0.11^	39.98	[-0.03, 0.25]	.147	8349	0.18	39.99	[0.10, 0.26]	< .001 ***
**Violence and conflict**															
Conflict & instability	8653	0.15	40.99	[0.07, 0.24]	.001 **	8562	0.05^	40.98	[-0.09, 0.19]	.454	8541	0.15	41.00	[0.07, 0.23]	< .001 ***
Homicide	8652	0.13^	41.02	[0.04, 0.22]	.008 **	8564	0.17	40.99	[0.04, 0.30]	.014 *	8544	0.13^	41.02	[0.05, 0.22]	.004 **
Political violence	8851	0.10^	42.01	[0.01, 0.19]	.031 *	8758	0.11^	42.00	[-0.02, 0.25]	.109	8741	0.11^	42.01	[0.02, 0.19]	.019 *
**Environmental and public health**															
Drought, floods & extreme temperatures	8253	-0.06^	39.03	[-0.15, 0.04]	.251	8173	-0.24	39.06	[-0.35, -0.13]	< .001 ***	8154	-0.07^	39.00	[-0.17, 0.02]	.148
Pathogen prevalence	8654	0.02^	41.00	[-0.07, 0.12]	.647	8557	-0.11^	40.98	[-0.24, 0.03]	.144	8541	0.04^	40.99	[-0.05, 0.14]	.388
Food insecurity	8454	-0.09^	40.00	[-0.19, 0.00]	.064	8368	-0.01^	39.98	[-0.16, 0.13]	.845	8344	-0.14^	39.99	[-0.22, -0.05]	.004 **
Childhood mortality	8454	0.02^	40.01	[-0.08, 0.12]	.706	8368	-0.04^	39.98	[-0.18, 0.10]	.589	8344	0.08^	40.00	[-0.01, 0.18]	.089
Life expectancy	8454	-0.06^	40.01	[-0.16, 0.04]	.227	8368	0.00^	39.99	[-0.15, 0.14]	.957	8344	-0.11^	40.00	[-0.20, -0.02]	.022 *
**Governance**															
Strength of democracy	8851	-0.05^	41.99	[-0.15, 0.04]	.261	8758	0.17	41.98	[0.04, 0.29]	.017 *	8741	-0.06^	41.98	[-0.15, 0.03]	.218
Political stability	8851	-0.15	42.00	[-0.23, -0.06]	.001 **	8758	-0.05^	41.99	[-0.18, 0.09]	.518	8741	-0.15	42.00	[-0.23, -0.07]	< .001 ***

Government effectiveness	8851	-0.20	42.02	[-0.28, -0.13]	< .001 ***	8758	-0.15	42.00	[-0.28, -0.02]	.028 *	8741	-0.20	42.00	[-0.27, -0.12]	< .001 ***
Rule of law	8851	-0.17	42.00	[-0.25, -0.09]	< .001 ***	8758	-0.07^	41.99	[-0.21, 0.07]	.325	8741	-0.16	41.99	[-0.24, -0.08]	< .001 ***
Corruption control	8851	-0.17	42.00	[-0.25, -0.10]	< .001 ***	8758	-0.11^	41.98	[-0.24, 0.03]	.132	8741	-0.17	41.99	[-0.25, -0.10]	< .001 ***
**Digital media landscape**															
Daily time spent on internet	8453	0.12^	40.01	[0.03, 0.21]	.010 *	8363	0.06^	39.99	[-0.08, 0.20]	.412	8346	0.14^	39.99	[0.05, 0.23]	.003 **
Daily time spent on social media	8453	0.11^	40.01	[0.02, 0.21]	.019 *	8363	0.03^	39.99	[-0.12, 0.17]	.690	8346	0.14^	39.99	[0.05, 0.22]	.003 **
Online media consistency	8851	-0.14^	42.02	[-0.22, -0.05]	.003 **	8758	-0.19	42.00	[-0.32, -0.06]	.006 **	8741	-0.12^	41.99	[-0.20, -0.03]	.012 *

Note. Each line indicates three separate LMM with country-level variables predicting the (1) perceived climate of polarization and (2) perceived anomie. Predictors have been standardized ($M = 0$, $SD = 1$) so beta values represent expected change in the outcome variable (in raw units) for one *SD* increase in the predictor. ^ indicates beta values below .15, which were estimated with lower precision given the available power. CI = confidence interval. * $p < .05$, ** $p < .01$, *** $p < .001$.

Supplementary Table 6. Bootstrapped Mediation Effect of All Country-Level Measures on the Perceived Climate of Polarization via Perceptions of Anomie

		Direct Effect				Indirect Effect (Leadership Breakdown)				Indirect Effect (Social Fabric Breakdown)				Total Effect			
**Predictor**	***N***	Est.	*SE*	95% CI	*p*	Est.	*SE*	95% CI	*p*	Est.	*SE*	95% CI	*p*	Est.	*SE*	95% CI	*p*
**Economy and inequality**																	
GDP PPP per capita	8422	-0.09	0.03	[-0.15, -0.04]	.001 **	-0.01	0.01	[-0.02, 0.00]	.150	-0.03	0.01	[-0.05, -0.01]	.003 **	-0.13	0.04	[-0.21, -0.06]	< .001 ***
Income inequality	7842	0.07	0.03	[0.01, 0.14]	.024 *	0.01	0.00	[0.00, 0.02]	.001 **	0.04	0.01	[0.02, 0.06]	< .001 ***	0.12	0.04	[0.05, 0.20]	.001 **
Unemployment	8618	0.08	0.05	[-0.02, 0.17]	.111	0.02	0.00	[0.01, 0.02]	< .001 ***	0.05	0.01	[0.03, 0.08]	< .001 ***	0.14	0.06	[0.02, 0.26]	.019 *
Youth NEET	8231	0.10	0.04	[0.03, 0.17]	.008 **	0.01	0.01	[0.00, 0.02]	.012 *	0.05	0.01	[0.04, 0.07]	< .001 ***	0.16	0.04	[0.08, 0.24]	< .001 ***
**Violence and conflict**																	
Conflict & instability	8425	0.11	0.02	[0.07, 0.15]	< .001 ***	0.01	0.01	[0.00, 0.02]	.065	0.05	0.01	[0.02, 0.07]	< .001 ***	0.17	0.03	[0.11, 0.22]	< .001 ***
Homicide	8422	0.07	0.03	[0.02, 0.13]	.009 **	0.01	0.00	[0.01, 0.02]	< .001 ***	0.04	0.01	[0.03, 0.05]	< .001 ***	0.13	0.03	[0.07, 0.18]	< .001 ***
Political violence	8618	0.06	0.02	[0.01, 0.11]	.010 *	0.01	0.00	[0.00, 0.02]	.021 *	0.03	0.01	[0.01, 0.05]	.004 **	0.10	0.04	[0.04, 0.17]	.003 **
**Environmental and public health**																	
Drought, floods & extreme temperatures	8032	-0.02	0.02	[-0.06, 0.02]	.395	-0.02	0.01	[-0.02, -0.01]	.002 **	-0.02	0.02	[-0.06, 0.01]	.134	-0.06	0.02	[-0.10, -0.01]	.012 *
Pathogen prevalence	8423	0.02	0.03	[-0.04, 0.08]	.512	-0.01	0.01	[-0.02, 0.01]	.380	0.01	0.02	[-0.02, 0.04]	.595	0.02	0.04	[-0.06, 0.11]	.606
Food insecurity	8229	-0.06	0.03	[-0.12, 0.01]	.071	-0.01	0.01	[-0.02, 0.00]	.263	-0.04	0.01	[-0.07, -0.01]	.003 **	-0.10	0.05	[-0.19, -0.01]	.023 *
Childhood mortality	8229	0.00	0.03	[-0.05, 0.05]	.875	0.00	0.01	[-0.01, 0.02]	.886	0.02	0.02	[-0.01, 0.05]	.133	0.02	0.04	[-0.06, 0.10]	.649
Life expectancy	8229	-0.03	0.03	[-0.10, 0.04]	.362	0.00	0.01	[-0.02, 0.01]	.555	-0.03	0.02	[-0.06, 0.00]	.037 *	-0.07	0.05	[-0.16, 0.03]	.163
**Governance**																	
Strength of democracy	8618	-0.05	0.03	[-0.11, 0.00]	.065	0.01	0.01	[0.00, 0.03]	.139	-0.01	0.02	[-0.06, 0.03]	.517	-0.06	0.05	[-0.16, 0.05]	.286
Political stability	8618	-0.11	0.03	[-0.17, -0.05]	< .001 ***	-0.01	0.01	[-0.02, 0.01]	.229	-0.04	0.01	[-0.07, 0.02]	.002 **	-0.16	0.04	[-0.24, -0.08]	< .001 ***
Government effectiveness	8618	-0.15	0.02	[-0.19, -0.10]	< .001 ***	-0.01	0.00	[-0.02, -0.01]	.002 **	-0.06	0.01	[-0.07, -0.04]	< .001 ***	-0.21	0.03	[-0.27, -0.16]	< .001 ***
Rule of law	8618	-0.13	0.03	[-0.18, -0.07]	< .001 ***	-0.01	0.01	[-0.02, 0.00]	.104	-0.05	0.01	[-0.07, -0.02]	.001 **	-0.18	0.04	[-0.26, -0.11]	< .001 ***

Corruption control	8618	-0.13	0.03	[-0.18, -0.08]	< .001 ***	-0.01	0.01	[-0.02, 0.00]	.023 *	-0.05	0.01	[-0.07, -0.03]	< .001 ***	-0.19	0.04	[-0.26, -0.12]	< .001 ***
**Digital media landscape**																	
Daily time spent on internet	8228	0.09	0.03	[0.02, 0.15]	.007 **	0.01	0.01	[0.00, 0.02]	.097	0.04	0.01	[0.02, 0.06]	< .001 ***	0.14	0.04	[0.06, 0.21]	< .001 ***
Daily time spent on social media	8228	0.08	0.04	[0.01, 0.15]	.025 *	0.01	0.01	[0.00, 0.02]	.160	0.04	0.01	[0.02, 0.07]	< .001 ***	0.13	0.04	[0.04, 0.21]	.003 **
Online media consistency	8618	-0.09	0.03	[-0.15, -0.03]	.002 **	-0.01	0.01	[-0.02, 0.00]	.005 **	-0.03	0.01	[-0.06, -0.01]	.013 *	-0.14	0.04	[-0.21, -0.06]	< .001 ***

Note. Bootstrapped estimates after 5000 simulations. All models control for demographic variables. Est. = estimate. CI = confidence interval. * $p < .05$, ** $p < .01$, *** $p < .001$.

Supplementary Table 7. Bootstrapped Mediation Effect of All Country-Level Measures on the Perceived Climate of Polarization via Perceptions of Anomie (Without Demographic Control Variables)

		Direct Effect				Indirect Effect (Leadership Breakdown)				Indirect Effect (Social Fabric Breakdown)				Total Effect			
**Predictor**	*N*	Est.	*SE*	95% CI	*p*	Est.	*SE*	95% CI	*p*	Est.	*SE*	95% CI	*p*	Est.	*SE*	95% CI	*p*
**Economy and inequality**																	
GDP PPP per capita	8717	-0.09	0.03	[-0.15, -0.03]	.002 **	-0.01	0.01	[-0.02, 0.01]	.274	-0.04	0.01	[-0.06, -0.01]	.001 **	-0.13	0.04	[-0.21, -0.05]	.001 **
Income inequality	8114	0.07	0.03	[0.01, 0.13]	.026 *	0.01	0.00	[0.00, 0.02]	.004 **	0.04	0.01	[0.03, 0.06]	< .001 ***	0.12	0.04	[0.05, 0.19]	.001 **
Unemployment	8917	0.08	0.05	[-0.02, 0.18]	.108	0.01	0.00	[0.01, 0.02]	< .001 ***	0.05	0.01	[0.03, 0.08]	< .001 ***	0.15	0.06	[0.03, 0.26]	.017 *
Youth NEET	8517	0.09	0.04	[0.02, 0.16]	.018 *	0.01	0.01	[0.00, 0.02]	.125	0.05	0.01	[0.03, 0.07]	< .001 ***	0.15	0.05	[0.06, 0.24]	.001 **
**Violence and conflict**																	
Conflict & instability	8715	0.10	0.02	[0.06, 0.15]	< .001 ***	0.00	0.01	[-0.01, 0.01]	.359	0.05	0.01	[0.02, 0.07]	< .001 ***	0.15	0.03	[0.09, 0.22]	< .001 ***
Homicide	8717	0.07	0.03	[0.02, 0.13]	.009 **	0.01	0.00	[0.01, 0.02]	< .001 ***	0.04	0.01	[0.03, 0.06]	< .001 ***	0.13	0.03	[0.07, 0.18]	< .001 ***
Political violence	8917	0.06	0.03	[0.01, 0.11]	.018 *	0.01	0.01	[0.00, 0.02]	.094	0.03	0.01	[0.01, 0.06]	.002 **	0.10	0.04	[0.03, 0.17]	.005 **
**Environmental and public health**																	
Drought, floods & extreme temperatures	8317	-0.02	0.02	[-0.06, 0.02]	.391	-0.02	0.01	[-0.03, -0.01]	< .001 ***	-0.02	0.02	[-0.06, 0.01]	.216	-0.06	0.02	[-0.10, -0.01]	.019 *
Pathogen prevalence	8716	0.02	0.03	[-0.04, 0.08]	.537	-0.01	0.01	[-0.02, 0.00]	.193	0.01	0.02	[-0.02, 0.04]	.409	0.02	0.04	[-0.06, 0.11]	.600
Food insecurity	8515	-0.05	0.04	[-0.12, 0.02]	.162	0.00	0.01	[-0.01, 0.01]	.825	-0.04	0.01	[-0.07, -0.02]	.001 **	-0.09	0.05	[-0.19, 0.01]	.062
Childhood mortality	8515	0.00	0.03	[-0.06, 0.05]	.887	0.00	0.01	[-0.02, 0.01]	.683	0.03	0.01	[0.00, 0.05]	.062	0.02	0.05	[-0.07, 0.11]	.673
Life expectancy	8515	-0.03	0.04	[-0.10, 0.04]	.459	0.00	0.01	[-0.02, 0.01]	.969	-0.03	0.01	[-0.06, -0.01]	.011 *	-0.06	0.05	[-0.16, 0.04]	.214
**Governance**																	
Strength of democracy	8917	-0.05	0.03	[-0.11, 0.01]	.081	0.01	0.01	[0.00, 0.03]	.051	-0.02	0.02	[-0.06, 0.03]	.424	-0.06	0.05	[-0.16, 0.05]	.297
Political stability	8917	-0.10	0.03	[-0.17, -0.04]	.003 **	0.00	0.01	[-0.02, 0.01]	.563	-0.05	0.01	[-0.07, -0.02]	.001 **	-0.15	0.05	[-0.24, -0.06]	.001 **
Government effectiveness	8917	-0.14	0.03	[-0.19, -0.09]	< .001 ***	-0.01	0.00	[-0.02, 0.00]	.009 **	-0.06	0.01	[-0.08, -0.04]	< .001 ***	-0.20	0.03	[-0.26, -0.14]	< .001 ***

Rule of law	8917	-0.12	0.03	[-0.18, -0.06]	< .001 ***	-0.01	0.01	[-0.02, 0.01]	.302	-0.05	0.01	[-0.07, -0.02]	< .001 ***	-0.17	0.04	[-0.25, -0.09]	< .001 ***
Corruption control	8917	-0.12	0.03	[-0.17, -0.06]	< .001 ***	-0.01	0.01	[-0.02, 0.00]	.107	-0.05	0.01	[-0.07, -0.03]	< .001 ***	-0.17	0.04	[-0.25, -0.10]	< .001 ***
**Digital media landscape**																	
Daily time spent on internet	8515	0.08	0.03	[0.01, 0.14]	.024 *	0.01	0.01	[-0.01, 0.02]	.355	0.04	0.01	[0.02, 0.07]	< .001 ***	0.12	0.04	[0.04, 0.21]	.003 **
Daily time spent on social media	8515	0.07	0.04	[0.00, 0.14]	.056	0.00	0.01	[-0.01, 0.01]	.630	0.04	0.01	[0.02, 0.07]	< .001 ***	0.12	0.05	[0.03, 0.21]	.013 *
Online media consistency	8917	-0.09	0.03	[-0.15, -0.03]	.003 **	-0.01	0.01	[-0.02, 0.00]	.009 **	-0.04	0.01	[-0.06, -0.01]	.010 *	-0.14	0.04	[-0.21, -0.06]	< .001 ***

Note. Bootstrapped estimates after 5000 simulations. Est. = estimate. CI = confidence interval. * $p < .05$, ** $p < .01$, *** $p < .001$.

Supplementary References

Chen, F. F. (2007). Sensitivity of goodness of fit indexes to lack of measurement invariance. *Structural Equation Modeling, 14*(3), 464–504. https://doi.org/10.1080/10705510701301834

Iyengar, S., Sood, G., & Lelkes, Y. (2012). Affect, not ideology: A social identity perspective on polarization. *Public Opinion Quarterly*, *76*(3), 405-431. https://doi.org/10.1093/poq/nfs038

Putnick, D. L., & Bornstein, M. H. (2016). Measurement invariance conventions and reporting: The state of the art and future directions for psychological research. *Developmental Review*, *41*, 71–90. https://doi.org/10.1016/j.dr.2016.06.004

Taiwanese Department of Household Registration. (January 9, 2025). Sex ratio in Taiwan from 2000 to 2024 (female=100) [Graph]. In *Statista*. Retrieved September 18, 2025, from https://www.statista.com/statistics/319808/taiwan-sex-ratio/

World Bank Group. (2024). *Population, Female (% of Total Population)* [Data set]. https://data.worldbank.org/indicator/SP.POP.TOTL.FE.ZS
