# Supplementary material for: Country-level instability is related to a stronger perceived climate of polarization across 44 countries
Source: Commun Psychol. 2026 Feb 25;4:63. doi: 10.1038/s44271-026-00422-x (PMC13046976; doi:10.1038/s44271-026-00422-x)
Supplement: Supplementary file 3 — Reporting Summary [file 44271_2026_422_MOESM3_ESM.pdf]

Corresponding author(s): Amy S. G. Lee
Last updated by author(s): Feb 2, 2026

Reporting Summary

Nature Portfolio wishes to improve the reproducibility of the work that we publish. This form provides structure for consistency and transparency in reporting. For further information on Nature Portfolio policies, see our Editorial Policies and the Editorial Policy Checklist.

Statistics

For all statistical analyses, confirm that the following items are present in the figure legend, table legend, main text, or Methods section.

n/a	Confirmed	
☐	☒	The exact sample size (*n*) for each experimental group/condition, given as a discrete number and unit of measurement
☐	☒	A statement on whether measurements were taken from distinct samples or whether the same sample was measured repeatedly
☒	☐	The statistical test(s) used AND whether they are one- or two-sided<br>*Only common tests should be described solely by name; describe more complex techniques in the Methods section.*
☐	☒	A description of all covariates tested
☐	☒	A description of any assumptions or corrections, such as tests of normality and adjustment for multiple comparisons
☐	☒	A full description of the statistical parameters including central tendency (e.g. means) or other basic estimates (e.g. regression coefficient) AND variation (e.g. standard deviation) or associated estimates of uncertainty (e.g. confidence intervals)
☐	☒	For null hypothesis testing, the test statistic (e.g. *F*, *t*, *r*) with confidence intervals, effect sizes, degrees of freedom and *P* value noted<br>*Give P values as exact values whenever suitable.*
☒	☐	For Bayesian analysis, information on the choice of priors and Markov chain Monte Carlo settings
☐	☒	For hierarchical and complex designs, identification of the appropriate level for tests and full reporting of outcomes
☐	☒	Estimates of effect sizes (e.g. Cohen's *d*, Pearson's *r*), indicating how they were calculated

Our web collection on statistics for biologists contains articles on many of the points above.

Software and code

Policy information about availability of computer code

Data collection	No software was used.
Data analysis	All data analyses were conducted in R.

For manuscripts utilizing custom algorithms or software that are central to the research but not yet described in published literature, software must be made available to editors and reviewers. We strongly encourage code deposition in a community repository (e.g. GitHub). See the Nature Portfolio guidelines for submitting code & software for further information.

Data

Policy information about availability of data

All manuscripts must include a data availability statement. This statement should provide the following information, where applicable:

- Accession codes, unique identifiers, or web links for publicly available datasets
- A description of any restrictions on data availability
- For clinical datasets or third party data, please ensure that the statement adheres to our policy

The data, codebook, and R code are available on the Open Science Framework: https://osf.io/pnbxr/overview?view_only=d01f2fb44e5643b192811b7e4b4c6e45.

Research involving human participants, their data, or biological material

Policy information about studies with human participants or human data. See also policy information about sex, gender (identity/presentation), and sexual orientation and race, ethnicity and racism.

Reporting on sex and gender	We had no sex- or gender-related hypotheses. However, self-reported gender was included as a covariate in our models. We report full question wording in the Supplementary Materials and in the codebook available on the Open Science Framework: https://osf.io/pnbxr/overview?view_only=d01f2fb44e5643b192811b7e4b4c6e45.
Reporting on race, ethnicity, or other socially relevant groupings	Our analyses included the following demographic measures: gender, age, religiosity, socioeconomic status, and political orientation on economic and social issues. These were included as covariates to control for potential confounding influences on the perceived climate of polarization. We report full question wording for these measures in the Supplementary Materials and in the codebook available on the Open Science Framework: https://osf.io/pnbxr/overview?view_only=d01f2fb44e5643b192811b7e4b4c6e45.
Population characteristics	See above.
Recruitment	Data were collected by an external survey company, Dynata, between May and June 2024. At least 200 participants were recruited from each of the 44 countries included in the study (N = 8917): Argentina, Australia, Austria, Belgium, Brazil, Bulgaria, Canada, Chile, China, Colombia, Czech Republic, Denmark, Finland, France, Germany, Greece, Hong Kong, Hungary, India, Indonesia, Ireland, Italy, Japan, Mexico, Netherlands, New Zealand, Norway, Philippines, Poland, Portugal, Romania, Singapore, Slovakia, South Africa, South Korea, Spain, Sweden, Taiwan, Thailand, Turkey, United Arab Emirates, United Kingdom, United States, and Vietnam.
Ethics oversight	The study received ethics approval from the University of Melbourne ethics committee (Project #28441).

Note that full information on the approval of the study protocol must also be provided in the manuscript.

Field-specific reporting

Please select the one below that is the best fit for your research. If you are not sure, read the appropriate sections before making your selection.

☐ Life sciences ☒ Behavioural & social sciences ☐ Ecological, evolutionary & environmental sciences

For a reference copy of the document with all sections, see nature.com/documents/nr-reporting-summary-flat.pdf

Behavioural & social sciences study design

All studies must disclose on these points even when the disclosure is negative.

Study description	Quantitative cross-sectional, multi-national study.
Research sample	Convenience samples of adults aged 18 years and over were recruited from 44 different countries (N = 8917) by Dynata, an external survey company: Argentina, Australia, Austria, Belgium, Brazil, Bulgaria, Canada, Chile, China, Colombia, Czech Republic, Denmark, Finland, France, Germany, Greece, Hong Kong, Hungary, India, Indonesia, Ireland, Italy, Japan, Mexico, Netherlands, New Zealand, Norway, Philippines, Poland, Portugal, Romania, Singapore, Slovakia, South Africa, South Korea, Spain, Sweden, Taiwan, Thailand, Turkey, United Arab Emirates, United Kingdom, United States, and Vietnam. Data were collected to ensure a relatively even gender split (4277 male; 4622 female; 14 other; 4 prefer not to say) and at least 200 participants were recruited per country. All participants passed the two attention check items and no participants were considered speeders based on the median response time and interquartile range.
Sampling strategy	The 44 countries were selected based on availability through Dynata. As such, the sample represents a convenience sample. Dynata recruited participants through pre-established panels in each country.
Data collection	Data were collected via an online questionnaire.
Timing	Data were collected between May and June 2024.
Data exclusions	No data were excluded from analyses.
Non-participation	No participants dropped out or declined participation.
Randomization	Participants were not allocated to experimental groups.

Reporting for specific materials, systems and methods

We require information from authors about some types of materials, experimental systems and methods used in many studies. Here, indicate whether each material, system or method listed is relevant to your study. If you are not sure if a list item applies to your research, read the appropriate section before selecting a response.

Materials & experimental systems

n/a	Involved in the study
☒	☐ Antibodies
☒	☐ Eukaryotic cell lines
☒	☐ Palaeontology and archaeology
☒	☐ Animals and other organisms
☒	☐ Clinical data
☒	☐ Dual use research of concern
☒	☐ Plants

Methods

n/a	Involved in the study
☒	☐ ChIP-seq
☒	☐ Flow cytometry
☒	☐ MRI-based neuroimaging

Plants

Seed stocks	N/A
Novel plant genotypes	N/A
Authentication	N/A
